# The Application of Nanotechnology in the Codelivery of Active Constituents of Plants and Chemotherapeutics for Overcoming Physiological Barriers during Antitumor Treatment

**DOI:** 10.1155/2019/9083068

**Published:** 2019-12-13

**Authors:** Qiushuang Li, Yang Xiong, Conghua Ji, Zhiqiang Yan

**Affiliations:** ^1^Center of Clinical Evaluation and Analysis, The First Affiliated Hospital of Zhejiang Chinese Medical University, Hangzhou 310006, China; ^2^Department of Pharmaceutical Science, Zhejiang Chinese Medical University, Hangzhou 310053, China; ^3^Institute of Biomedical Engineering and Technology, Shanghai Engineering Research Center of Molecular Therapeutics and New Drug Development, School of Chemistry and Molecular Engineering, East China Normal University, Shanghai 200062, China

## Abstract

Antitumor therapy using a combination of drugs has shown increased clinical efficacy. Active constituents derived from plants can offer several advantages, such as high efficiacy, low toxicity, extensive effects, and multiple targets. At present, the combination of plants' active constituents and chemotherapeutic drugs has attracted increased attention. Nanodrug delivery systems (NDDSs) have been widely used in tumor-targeted therapy because of their efficacy of delivering antitumor drugs. The *in vivo* process of tumor-targeted NDDSs has several steps. They include blood circulation, tumor accumulation and penetration, target cell internalization and uptake, and drug release and drug response. In each step, NDDSs encounter multiple barriers that prevent their effective delivery to target sites. Studies have been performed to find alternative strategies to overcome these barriers. We reviewed the recent progress of codelivery of active constituents of plants and chemotherapeutics using NDDSs. Progress into transversing the physiological barriers for more effective *in vivo* antitumor delivery will be discussed in this review.

## 1. Introduction

Cancer is one of the most deadly diseases that endangers human health. Chemotherapy is currently the major treatment strategy for treating cancers and preventing postsurgical recurrence. However, multidrug resistance (MDR) in tumor cells and serious adverse effects have hindered chemotherapy [1]. To address these issues, studies have been performed to investigate the effects of drug combinations for cancer treatment. The combination of active constituents of plants with first-line chemotherapy drugs has shown good efficacy in reversing tumor chemoresistance, enhancing curative effects, and reducing adverse reactions. Combination treatment of active constituents of plants with chemotherapy drugs for tumor therapy has recently become very popular [2–4]. However, direct administration of free drugs has several disadvantages, such as short duration in blood circulation and nonselectivity for tumor tissue and tumor cells. This reduces efficacy while increasing adverse reactions due to nonspecific targeting of healthy tissue. To solve this problem, several strategies have been developed. Nanodrug delivery systems (NDDSs) have demonstrated potential advantages for cancer therapy. The most common carriers of NDDSs include liposomes, nanoparticles, micelles, and polymers. They can effectively increase the duration of drugs in systemic circulation, improve pharmacokinetics, and promote drug tumor targeting and tumor accumulation. All these substantially increase the curative effects while reducing toxicity [5, 6]. Intravenous administration of NDDSs results in a series of complex *in vivo* delivery processes, which includes blood circulation, tumor targeting, tumor accumulation, tumor tissue penetration, tumor cell internalization, and intracellular transport. Several specific drug delivery barriers exist, with each directly affecting efficacy. In order to improve drug efficacy and reduce adverse reactions of NDDSs, researchers have developed several exceptional delivery strategies to overcome these barriers. In this review, the physiological basis of designing tumor-targeted drug delivery systems to overcome these physiological barriers will be discussed.

## 2. Tumor Pathophysiology

The pathophysiological features of the tumor are the basis for designing tumor-targeting drug delivery systems [7]. One of the important physiological features of tumor tissues is their enhanced permeability and retention effect (EPR effect) to nanoparticles. Tumors that reach greater than 2 mm^3^ are highly dependent on nutrients and oxygen that are supplied by tumor blood vessels. Tumor and lymph angiogenesis start to develop when tumor blood vessels are unable to meet the requirements of the rapidly growing tumor [8]. Blood vessels that have recently formed through neovascularization have enhanced permeability, lack a smooth muscle layer, and has dysfunctional angiotensin receptors. In addition, lymph vessels in the center of tumor tissues are usually dysfunctional, which results in lymphatic obstruction and retention of macromolecular substances like lipid particles. The high selective permeability and retention in tumor tissues are termed the EPR effect [9]. The EPR effect is the basis for designing passive tumor targeting NDDSs [10].

Additionally, unlike normal cells, tumor cells grow in an uncontrolled and invasive manner. In order to infinitely proliferate, tumor cells have increased expression of certain receptors. These include the folate receptor (FR) [11], integrin receptor, transferrin receptor (TfR), somatostatin receptor, vasoactive intestinal peptide receptor, and cholecystokinin receptor. In addition, several specific receptors are expressed on the surface of tumor blood vessels, such as vascular endothelial growth factor (VEGF) receptor [12], integrin *α*v*β*3 [13], and E-Selectin [14]. Many of these receptors that are overexpressed are common in tumor tissue and tumor blood vessels. The active targeting mechanism of NDDS relies on these specific receptors to bind specifically to tumors. However, long-term administration of antitumor drugs induces P-glycoprotein (P-gp) overexpression in tumor cells. P-gp functions to expel antitumor drugs from tumor cells, thus reducing the intracellular drug concentration, which in-turn reduces antitumor efficacy and makes tumor cells resistant to chemotherapy. This process is termed MDR [15]. MDR has been identified in almost all human tumor cells.

Cancer stem cells (CSCs) in tumor tissues [16] have the ability to self-renew, multiply, and differentiate. They can also stimulate the growth of new tumors [17]. Even though they exist in limited numbers, CSCs play a significant role in the development, progression, metastasis, and recurrence of tumors. Conventional chemotherapy or radiotherapy induces tumor cell death to reduce tumor cell numbers and prevent the rapid growth of tumors [18]. However, CSCs are not sensitive to conventional chemotherapy or radiotherapy and are not completely eliminated [19, 20]. CSCs are one of the main reasons for tumor recurrence.

Tumor cells have a very high metabolic rate and produce high levels of acid leading to an acidic environment in tumor tissues. The extracellular pH in tumor tissues is approximately 6.0–6.5. In addition, the reduced blood supply to the central area of the tumor leads to local hypoxia to increase the acidic environment [21]. Tumor tissues also have several physiological features that include high interstitial fluid pressure, specific enzymes, and oxidative stress [22].

## 3. Types of Tumor-Targeted NDDSs for Plant Chemotherapeutic Drugs

Currently, the most commonly used NDDSs include liposomes, nanoparticles, polymeric micelle, and products of polymer-drug conjugates. The major structural features and drug-carrying mechanisms of these NDDSs are listed in Table 1.

### 3.1. Liposome

Liposomes are lipid nanovesicles formed by lipid bilayers [23–25]. The diameter of a liposome is approximately 90–200 nm. The center of the liposome consists of a hydrophilic internal aqueous phase. The internal aqueous phase and lipid bilayer of the liposome could be used to carry a variety of cargos. For instance, hydrophilic drugs could be packed into the internal aqueous phase, while hydrophobic drugs could be packed into the lipid membrane. Additionally, amphiprotic drugs could be packed in the aqueous phase and phospholipid membrane. Furthermore, antibodies and polypeptides could be used to modify the surface of liposomes to make them to target various organs or tumors [23]. Liposomes continuously release their loaded drugs slowly and hence have the ability to change the distribution and pharmacokinetic properties of the drugs, thereby reducing their toxic effects [24]. Long circulating liposomes will add significant benefit for long-term drug delivery but needs to be further optimized.

In general, the surface of liposomes is modified by hydrophilic macromolecules, such as polyethylene glycol (PEG), to reduce their recognition by opsonin in the blood and to reduce phagocytosis by the reticuloendothelial system (RES) which then increases the drug duration in blood circulation [25, 26]. There is a difference in water solubility between antitumor active constituents of plants and chemotherapeutic agents. This makes liposomes the preferred carrier for *in vivo* delivery of such drugs. To date, numerous studies have used liposomes as nanocarriers for combined antitumor drug therapy using active constituents of plants and chemotherapeutic agents. Hu et al. [27] developed a liposome using distearoylsn-glycero-3-phosphoethanolamine-*N*-(methoxy(polyethylene glycol)-2000) (DSPE-PEG 2000), which cocarried temozolomide (TMZ) and quercetin (QUE) for the treatment of drug-resistant U87 glioma cells. Transmission electron microscopy demonstrated that nanoliposomes loaded with TMZ and QUE had reduced diameters. *In vitro* studies demonstrated that this liposome could favor cellular uptake of drugs and thus effectively reduce the drug dose without reducing efficacy.

### 3.2. Nanoparticles

Nanoparticles are colloidal particles made from natural or synthetic high molecular polymers as carriers. The drugs are attached to the carrier material by physical entrapment, absorption, or chemical covalent binding. The natural high molecular polymers mainly include heparin [28], chitosan [29], gelatin [30], and albumin [31], while synthetic high molecular polymers are mainly polylactic acid (PLA), poly(lactic-co-glycolic acid) (PLGA), and polycaprolactone (PCL). Nanoparticles can be easily modified to increase their targeting capability. Compared with liposomes, nanoparticles have several advantages, such as better physical stability and higher drug-loading capability. In addition, they are easy to prepare, have certain degrees of sustained release function, and are suitable for packing insoluble drugs. Currently, nanoparticles have been used as carriers for targeting and controlled drug release and are administered by intravenous injection or topical administration. Xu et al. [32] developed a PLGA nanoparticle for the codelivery of docetaxel and gambogic acid. They identified the best ratio of docetaxel with gambogic acid, which was then packed into PLGA nanoparticles. Cell apoptosis and immunoblotting demonstrated that this codelivery nanoparticle could downregulate the expression of P-gp to increase cell apoptosis and thus effectively inhibit MDR of tumor cells.

### 3.3. Polymeric Micelles

Polymeric micelles are flexible spherical particles that are synthesized from the self-assembly of two amphiphilic block copolymers at the appropriate temperature and concentration. Polymers could be AB-type diblock copolymers or ABA-type triblock copolymers. Micelles formed using the triblock copolymer are more stable. Polymeric micelles have a hydrophobic core that is used to pack drugs that have poor water solubility or hydrophobic drugs. This increases the stability of the packed drugs and prevents their rapid degradation [33]. The hydrophilic outer shell forms a protective barrier to prevent the binding of micelles with plasma proteins. This prevents phagocytosis of the micelle by RES and thereby increases the half-life of the drug [34].

Yao et al. [35] designed a micelle that could codeliver paclitaxel (PTX) and curcumin (CUR) and demonstrated that it had a superior drug-loading capacity of up to 35% (w/w) and was able to synergistically induce anticancer effects. Sarisozen et al. [36] developed a mixed micelle using TF-polyethylene glycol polyethylene (TF-PEG-PE) that actively targeted TF to improve tumor targeting. Fang et al. [37] developed a magnetic micelle for the codelivery of doxorubicin and CUR. This codelivery system targeted lactoferrin and prolonged retention of the drugs at the tumor sites to efficiently suppress cancer growth compared with delivery of either drug alone.

### 3.4. Polymer-Drug Conjugates

In contrast to other drug-delivery systems, polymer-drug conjugates are drugs conjugated to a polymer via covalent bonding. This drug-delivery system has several advantages: (1) it increases the water solubility of hydrophobic drugs; (2) the covalent bonds could be modified (such as having a pH-sensitive linker, enzyme sensitive linker, or light sensitive linker). This enables the release of drugs to different sites [38]; (3) conjugation of drugs with polymers could increase their half-life; and (4) the increase in molecular weight could prevent EPR effect, thus favoring drug accumulation in solid tumors. Hence, these conjugates could overcome the differences in water solubility, selectivity, and stability between the antitumor active constituents of plants and chemotherapeutic drugs. The most commonly used polymers include polysaccharides, PEG, poly amino acids (PAA), and polypeptides [39].

Xue et al. [40] constructed a self-assembled prodrug nanoparticle that was conjugated to cabazitaxel and citronellol via a disulfide bond. This nanoparticle was redox-sensitive to high concentrations of glutathione (GSH) within tumor cells. Zhang et al. [41] designed a PEG-doxorubicin (DOX)-CUR prodrug nanoparticle for codelivery of DOX and CUR. Schiff-base reaction was used to conjugate DOX to PEG and then was conjugated to CUR in the nanoparticle to obtain PEG-DOX-CUR nanoparticles (NPs). The nanoparticle was acid-sensitive and hence could release DOX and CUR when it reached the tumor.

## 4. Strategies to Overcome the Physiological Barriers of NDDSs

**Figure 1:**
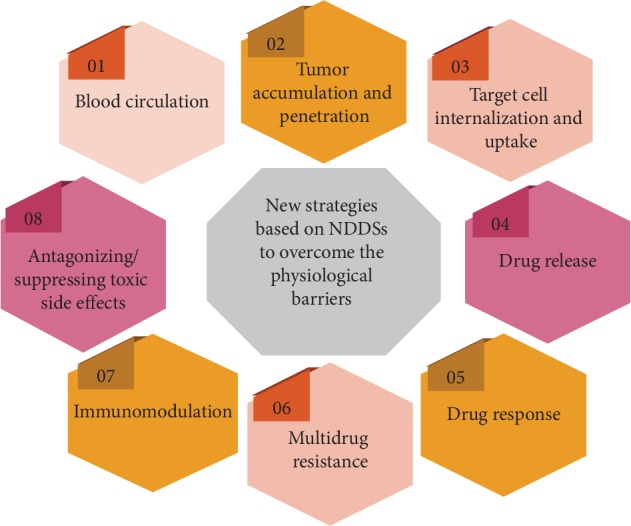
Development of new strategies based on NPs technology for drug delivery to overcome the transport barriers.

To effectively deliver drugs to target tissues and sites, NDDSs need to overcome a number of physiological and physical barriers. These include the blood, tissue, and cellular and intracellular transportation barriers. Each barrier could directly affect the final efficacy of the antitumor drugs. In order to overcome these barriers, researchers have developed a series of strategies and methods (see Figure 1 and Table 2).

### 4.1. Blood Circulation

After intravenous injection, NDDSs enter the blood circulation and encounter a number of obstacles: (1) degradation of the nanoparticles or drugs by the various enzymes in the plasma [95]; (2) opsonization of the nanoparticles and subsequent phagocytosis and clearing by RES [96, 97]; and (3) absorption of the nanoparticles by plasma proteins leading to the aggregation and subsequent hematotoxicity, retention in the pulmonary capillaries, or phagocytosis by RES [98]. To overcome these barriers, NDDS needs to avoid interactions with charged proteins in the blood or avoid phagocytosis. To overcome the limitations of NDDS in blood circulation, numerous studies have focused on modifying the surface of these nanoparticles. PEG coated to the surface of nanoparticles increases the half-life of nanoparticles [99]. It works by inhibiting the formation of the hydrophilic shells around NDDSs [100], which prevents plasma proteins from interacting with the nanoparticle and hence prevents RES. Yu et al. [42] developed a PEG-modified long-circulating liposome that concurrently packed QUE (p-gp inhibitor) and Adriamycin (AMD/DOX). This increased the blood concentration of AMD and half-life of AMD in the plasma. PEG-modified nanoparticles, PEG-modified grafted polymers, PEG-modified polymeric micelles, and PEG-modified dendrimers all have been shown to increase the half-life of drugs in blood circulation [43–47, 101].

However, several studies also observed that continuous injection of PEG-modified nanoparticles could induce immune responses and thus lead to accelerated blood clearance (ABC) [102, 103]. Hence, the design of drug-delivery systems with biological characteristics to evade the immune system has garnered increased attention [104]. Several studies have demonstrated that nanoparticles coated with endogenous substances like red blood cell membranes (RBC-NP) could avoid phagocytosis by macrophages via immunomodulatory proteins (such as CD47) on their surface and hence extend their half-lives [105].

### 4.2. Tumor Accumulation and Penetration

The low efficiency of drug accumulation in tumor tissues is one of the hurdles in antitumor NDDS therapy. NDDS accumulation in tumors is mainly via EPR. However, the dense extracellular matrix and the extremely high interstitial fluid pressure in tumors significantly prevent drugs from entering the deep tissues of the tumor. Because of this, the majority of the NDDS are distributed in blood vessels around the tumors. This results in lower drug distribution into the actual tumor, reduced efficacy, and hence an incomplete elimination and subsequent recurrence [27, 106]. Several studies have demonstrated that nanodrugs with smaller diameters have increased efficacy because they can penetrate easier and deeper into tumor tissues [33, 51].

Numerous studies have demonstrated that several specific receptors are expressed on the surface of tumor blood vessels compared with normal blood vessels. These include endothelial cell surface-specific receptors integrin*α*v *β*3 [107] and nucleolin [108]. NDDSs that are modified on the surface to express ligands to these receptors could target both tumor blood vessels and tumor cells. Targeting of tumor blood vessels could increase the retention of the NDDSs near the tumor, which eventually increases the distribution in tumor tissues [93]. For example, RGD peptide is a short peptide containing arginine, glycine, and aspartic acid (Arg-Gly-Asp) and is the ligand for integrin *α*v*β*3. Jiang et al. [48] developed an RGD-modified PTX and CUR coloaded liposomes and demonstrated better antitumor efficacy compared with unmodified liposomes. As a P-gp inhibitor, CUR could inhibit multidrug resistance, while the combined application of CUR and PTX had a synergistic effect. These RGD-modified nanoparticles have shown high aggregation and deep permeability at tumor sites [48–50]. Hence, targeting common receptors on tumor blood vessels and tumor cells is an effective strategy to improve the accumulation and penetration of nanoparticles into tumor tissues [52, 62, 109].

### 4.3. Target Cell Internalization and Uptake

After penetrating deep into tumor tissue, NDDS must be internalized by the tumor cells to exert into antitumor effects. Codelivery of active constituents of plants and chemotherapy drugs could promote NDDSs internalization by receptor-mediated endocytosis. In addition, P-gp inhibitors could be used to overcome the drug resistance in tumor cells [110–113].

Specific receptors that are highly expressed in tumors and on the surface of tumor endothelial cells include transferrin receptor, folate (FA) receptor, integrin receptor, somatostatin receptor, lectin receptor, and epidermal growth factor receptor (EGFR). NDDSs modified with different ligands or antibodies which is so-called active targeting NDDSs have shown better tumor-targeting and drug-delivery capability both *in vivo* and *in vitro*. TF and FA are very commonly used malignant tumor-targeting ligands, as most tumor cells express high levels of TfR or FR on their surface, while the expression of TfR and FR in normal tissues are much lower [114–116]. Cui et al. [55] used transferrin-modified nanoparticles for the codelivery of DOX and CUR. They first synthesized a pH-sensitive Tf-PEG-CUR prodrug and then packed DOX into the Tf-PEG-CUR NPs to obtain the Tf-PEG-CUR/DOX nanoparticle. This nanoparticle had active tumor-targeting features and could release their payload into tumor sites specifically because of its pH sensitivity in tumor tissue. Singh et al. [56] conjugated planetary ball-milled (PBM) nanoparticles and loaded it with resveratrol and DTXto FA for the treatment of prostate cancer. They found that Fa-modified DTX nanoparticles had increased cytotoxicity and hence could reduce the concentration of free drug by 28 folds. In addition, when DTX-resistant prostate cancer (PCa) cells were treated using this nanoparticle, there was a reversal of multidrug resistance in these cancer cells [57]. In addition, anti-GLUT1 antibody (GLUT1) has been used to modify polymeric micelles coloaded with CUR and doxorubicin and have shown increased efficacy in human colorectal cancer cell HCT-166 both *in vitro* and *in vivo* [58].

P-gp is a highly expressed multidrug transporter that could lead to MDR and chemotherapy failure in a number of cancers [15]. Numerous studies have demonstrated that the active constituents of plants, such as CUR, QUE, and triptolide, could inhibit the expression of P-gp gene and protein [117–121]. Thus, codelivery of such drugs with chemotherapeutic drugs could reverse MDR, increase chemotherapy sensitivity, and decrease adverse effects [53, 54]. Compared with chitosan-conjugated PLGA nanoparticles [122, 123], chitosan and anti-P-gp antibody-conjugated PLGA nanoparticles have demonstrated enhanced cell internalization. Hence, NDDS-targeting P-gp has provided an effective method to overcome drug resistance and increase drug internalization.

### 4.4. Drug Release

Drug release is the last step in drug delivery. Only released drugs from these nanoparticles can exert antitumor effects. Studies have taken advantage of the differences between the tumor microenvironment and the normal environment to figure out ways to release drugs from these nanoparticles. This has mainly been via dissociation of chemical bonds or structures that are sensitive to the tumor microenvironment [124, 125]. There are three common strategies for designing NDDS to promote drug release, i.e., pH-sensitive NDDSs, enzyme-sensitive NDDSs, and temperature-sensitive NDDSs.

The pH in the extracellular tumor tissues (∼pH 6.5) and endosomes/lysosomes (pH 4.5–6.0) are lower compared with normal tissues and blood (pH 7.4). This difference in pH could be used to design pH-responsive nanocarriers [126]. Acetal, hydrazine, imine, and esters are unstable at low pH and could be used to construct pH-responsive NDDSs. Xie et al. [59] utilized Schiff-base reaction to link methotrexate(MTX) with (DSPE-PEG2000) using a pH-sensitive imine linkage to obtain a pH-sensitive prodrug, i.e., DSPE-PEG-Imine-MTX. This prodrug could self-assemble to micellar nanoparticles (MTX-Imine-M) in aqueous solutions and could pack CUR into its core via hydrophobic interaction to form MTX-Imine-M-CUR nanoparticles. The active form of MTX is released more efficiently when the pH is 5.0 compared with 7.4. These nanoparticles are more efficacious and have lower toxicity profiles. In addition, pH-sensitive inorganic substances and polymers have been used to create nanoparticles to release drugs into tumor cells. pH-responsive nanocarriers, such as liposomes, nanoparticles, nanogels, polymer-drug conjugates, and micelle, have been designed and reported to have good efficacy and function [127]. Inorganic substances like calcium phosphate, chitosan, closed mesoporous silica nanoparticle pores (MSNPs), and poly(styrene-co-*N*,*N*′-dimethylaminoethyl methacrylate nanoparticles (P(St-co-DMAEMA))complexes and mPEG-b-PMaIPG (methoxy-polyethylene glycols (PEG)-b-poly (d-galactopyranose)) nanoparticles have been demonstrated to be sensitive to relatively small pH changes and have good drug release and relatively high antitumor activity [44, 49, 60, 62, 63]. Yang et al. [61] synthesized the pH-sensitive polymer (poly(ethylene glycol)-benzoic imine-poly(gamma-benzyl-l-aspartate)-b-poly(1-vinylimidazole) block copolymer (PPBV) and developed a multistage pH-responsive micelle system for the codelivery of PTX and CUR for the treatment of breast cancer CSCs. This multistage pH-responsive micelle system could intelligently convert the charges on the surface from neutralities to cations, reduce its diameter size to favor long-term blood circulation into tumor blood vessels, and promote tumor cell uptake and tumor permeability. All this enhanced the treatment efficacy of the nanoparticle for combination therapy using PTX and CUR.

Enzyme-responsive NDDS utilizes the overexpression of various enzymes in tumors to develop NDDS that contain substrates that could be specifically degraded by such enzymes [128]. Various proteases, such as matrix metalloproteases (MMPs) [129, 130] in tumor tissues and cathepsin in lysosomes of tumor cells [131, 132], are overexpressed in tumors. Li et al. [64] developed a protein NDDS that could intelligently respond in the tumor microenvironment. Based on the microenvironment, i.e., enzymes on the tumor surface (MMPs), pH, and high tumor GSH concentrations, the nanoparticle can respond and efficiently target tumors and also reduce metastatic rates.

Temperature-sensitive NDDS regulates drug release based on changes in temperature. Due to severe inflammatory reactions, the internal temperature of most solid tumors is generally higher compared with surrounding normal tissues. Temperature-sensitive drug-delivery systems could enhance the release of drugs in tumors, i.e., after adding the active tumor targeting feature, the temperature-sensitive targeting NDDS could be constructed [133]. Nguyen et al. [65] synthesized a heat-responsive Hep-F127 polymer for the codelivery of cisplatin (CDDP) and nano curcumin complex/pack to form a dual drug-delivery system. This nanoparticle had antiproliferative effects and tumor inhibition on MCF-7 cells and xenograft transplantation models. Zhang et al. [66] developed a near-infrared-responsive gold nanocagesusing biotin PEG thiol (biotin-PEG-SH) to codeliver doxorubicin and QUE for the treatment of breast cancer. This system had the feature of rapid drug release upon radiation of near-infrared rays and high cytotoxicity for MCF-7/ADR cells.

### 4.5. Drug Response

#### 4.5.1. Synergistic Antitumor Effects

Using a single chemotherapeutic drug for antitumor treatment has several limitations, such as inducing drug resistance, high toxicity, and low therapeutic index [75, 134]. Drug combinations for the treatment of cancers have become more favorable. Active constituents of plants have multiantitumor effects. They can synergistically inhibit tumor-cell proliferation by enhancing tumor-cell apoptosis, induce cell autophagy, enhance oxidative stress, improve the sensitivity, and increase cell cycle arrest when used in conjunction with chemotherapy drugs [69, 134–138]. Investigators have developed novel combinatory approaches using cationic PEGylatedniosome-encapsulated nanoparticles. These have demonstrated synergistic effects in gastric, prostate, and breast cancer cells [81]. Delivering nanoparticles (functional polymeric micelles, polyamidoamine dendritic polymers, and copolymers) that carry active constituents of plants and chemotherapeutic drugs is more efficacious compared with monotherapy [33, 35, 69–80, 82, 106].

#### 4.5.2. CSC-Targeting System

Although CSCs account for only a small portion of tumor cells, they have the capacity to self-renew, differentiate, and maintain tumor growth [139, 140]. CSCs can induce recurrence, metastasis, and resistance to antitumor drugs [141], which subsequently leads to chemotherapy failure. As the “drug pumps,” ABCG2 is highly expressed in CSCs. They pump drugs out of cells to prevent tumor killing. CSCs are considered the key to eradicating tumors. Signaling pathways and specific markers in CSCs could be the ideal targets for CSC-targeting NDDSs [142]. The active constituents of plants, such as CUR, can inhibit several signaling pathways, such as the Wnt/*β*-catenin, Notch, and Hedgehog pathways [143] and thus effectively inhibit the self-renewal of CSCs. Combining CSC-targeting therapy with conventional chemotherapy drugs could result in synergistic antitumor activity [61, 68]. For instance, pH-sensitive nanoparticles coloaded with CUR and DOX (CURDOX-NPs) prepared using monomethoxy (polyethylene glycol)-b-P (D, L-lactic-co-glycolic acid)-b-P (L-glutamic acid) polymer (mPEG-PLGA-PGlu) have shown better breast cancer-inhibitory effects compared with monotherapy [67].

#### 4.5.3. Multifunctional Targeting Drug Delivery Systems

To further increase the targeting of NDDS to tumor tissues, several multifunctional targeting drug delivery systems using different modifications to overcome multiple barriers simultaneously have been developed [86, 87]. Nanoplatform for combinational therapy using PEG, PCEC (poly(“-caprolactone)-b-poly(ethylene glycol)-b-poly(”-caprolactone)) and CRGDK (cell-penetrating peptide (Cys-Arg-Gly-Asp-Lys, CRGDK)) has been developed. These can coload DOX/CUR, target the tumor, and respond to intracellular acidic environments. The synergistic antitumor effects of these nanoparticles have the following four aspects: (1) increased stability in blood circulation; (2) passive targeting due to EPR; (3) active targeting by recognition of CRGDK to neuropilin-1 receptor; and (4) high stability and low drug leakage under physiological pH, while the acidic environment dissociates the prodrugs to release DOX and CUR into the cells [83]. Based on the expression of Rf in the blood-brain barrier and glioma cells, human TfR ligand T7 (sequence: HAIYPRH)-modified magnetic PLGA nanoparticle (MNP/T7PLGA NPs) target tumors and release PTX and CUR. This system provides a dual-targeting strategy, i.e., ligand-mediated targeting and magnetic-guided targeting [37, 84]. Saha et al. [85] used the high-temperature solvothermal technique to manufacture Eu : Gd_2_O_3_ triangular nanoplates. Using this method, nanoparticles are conjugated on its surface to FA, which is the targeting ligand and is then loaded with daunorubicin and CUR via ester bonds. The acidity in tumor tissues induces esterolysis to release the chemotherapeutic drugs into the tumor.

To enhance the antitumor effects of NDDSs, combination drug administration, CSCs-targeting drug delivery system, and multifunctional targeting drug delivery systems have been widely used to achieve additive or synergistic antitumor effects. This approach is a new promising method for efficient tumor targeting.

### 4.6. Multidrug Resistance

**Figure 2:**
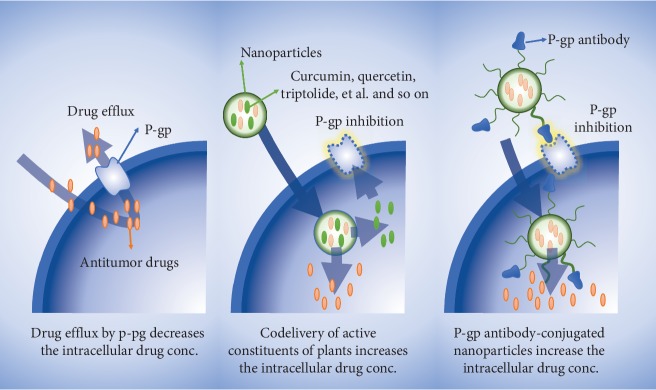
NDDS-mediated P-gp inhibition is an effective method to reverse MDR and increase drug internalization.

MDR occurs when tumor cells are resistant to one or a series of chemotherapeutic drugs with different structures and mechanisms. MDR is an important reason for chemotherapy failure in clinical practice [144, 145]. The mechanisms of MDR are very complex and include inherent cellular or changes in tumor microenvironments. The complexity of the mechanisms involving MDR brings about challenges to overcome tumor drug resistance [146, 147]. One of the advantages of using nanocarriers to codeliver chemotherapy drugs and active constituents of plants is their capability to reverse MDR [32, 91, 148](seen Figure 2). Rejinold et al. [88] investigated using CUR as the nanocarrier to load PEG-doxorubicin hydrochloride for HCT-8/DOX-resistant cells to increase the *in vivo* and *in vitro* antitumor efficacy. *In vitro* anti-MDR experiments have shown that PEG CRC/DOX NPs had a higher antimetastatic and antiproliferative effect on MDR cancer cells while normal fibroblasts were unaffected. In addition, PEG CRC/DOX NPs have longer blood circulation times compared with CRC NPs. Previous studies have used NDDS coloaded with PTX and active constituents of plants (such as baicalin and borneol) for anti-MDR. These *in vitro* experiments have shown that such combinations enhanced the concentration of PTX in MCF-7/Tax and A2780/PTX cells, as well as increased cellular drug and cytotoxic effects [89, 90].

### 4.7. Immunomodulation

The body's immune system has a significant influence on tumor development and progression. The tumor can modulate the immunocompetence of the body to recognize and kill tumor cells [149]. Quagliariello et al. [92] demonstrated that coadministration of rapamycin and QUE could reduce the levels of IL-8, IL-6, and IL-19, suggesting that such combinations could modulate the body's immune system and thus enhance the tumor-killing capability. In addition, such combinations could downregulate VEGF, MMP2, and MMP9 levels, suggesting they could inhibit tumor metastasis. Sesarman et al. [93] developed a long term circulating liposome that could pack CUR and PTX (LCL-CURC-DOX). This liposome could significantly increase the cytokine rations of IL-12/IL-4, IL12/IL-1*α*, IL-12/IL-1*β*, IFN-*γ*/IL-6, IFN-*γ*/IL-1*α*, and IFN-*γ*/IL-1*β* by 1.18–3.14-fold, (*P* < 0.05), thus favoring the balance of Th1 and Th2 cells to stimulate antitumor effects in the tumor microenvironment.

### 4.8. Antagonizing/Supressing Toxic Side Effects

Chemotherapy drugs damage normal tissues and cells in the body. Using plants (crude drugs) in combination with chemotherapy drugs could affect tumor tissues via multiple targets and pathways thereby reducing the dose of chemotherapy drugs and hence decrease drug toxicity. In addition, some plants (crude drugs) could also suppress the toxic side effects of chemotherapy. Hence, the combined application of such plants could increase the safety of chemotherapy. Guo et al. [150] combined andrographolide, the active constituent of the plant *Andrographis panicula*, with bleomycin and found that it not only enhanced the antitumor effects but also reduced the toxic effects of bleomycin on the body. In addition, the combined application of andrographolide and bleomycin effectively reduced pulmonary fibrosis induced by bleomycin, which was manifested by the activation of superoxide dismutase, and inhibition of malondialdehyde and hydroxyproline. Such combinations also suppressed cytokine expression. Zhang et al. [94] developed a complex polymeric micelle system that copacked Adriamycin and CUR (CPMDC) and investigated their protective effects on Adriamycin-induced cardiac toxicity. Pharmacokinetics and tissue distribution showed that CPMDC increased DOX accumulation in tumors but decreased the levels of the toxic metabolite doxorubicin in heart tissue compared with DOX alone.

## 5. Future Prospects

With the continuous advancements in the understanding of the mechanisms of tumor development and progression, strategies using combination drug therapy have demonstrated significant advantages for cancer treatment. The development of nanotechnology further provides broad application prospects. In this review, we summarized novel strategies and methods in developing NDDSs for codelivery of active constituents of plants and chemotherapy drugs to overcome barriers to drug delivery. NDDSs must circumvent all these barriers before successful and effective antitumor activity is observed. Any problems in these delivery systems could lead to tumor treatment failure. Hence, NDDSs should be appropriately designed based on the physiological process in the body to overcome these barriers. However, the preparation of multifunctional and intelligent tumor-targeted NDDSs is very complex, and hence reduces the drugability of NDDSs. Currently, NDDSs for codelivery still faces a lot of challenges regarding its formulation, design, synthesis, and assessment. More studies are needed to further investigate the pathological features of tumors. This could help in developing multifunctional NDDSs that response to the pathological features of the body. The combination of pharmaceuticals, medicine, chemistry, and materials science will help in the development of NDDSs.

## Figures and Tables

**Table 1:** Types and characteristics of nanodrug delivery systems.

Type	Structure	Drug loading	Advantages	Limitations
Liposomes	Lipid bilayer	Physical entrapment	Great biocompatibility, no immunogenicity	Low stability, hydrophilic drug easily leaks out
Nanoparticles	Nanosphere-/nanocapsule-/polymer-based nanoparticles with a lipophilic core	Physical encapsulation/chemical bonds	High drug-loading capability	—
Polymeric micelles	Core-shell structure formed by self-assembly	Physical packing/chemical bonding	Easy to prepare, increased stability of hydrophobic drugs	Low stability, depolymerizes after dilution
Polymer-drug conjugates	Conjugation of drugs with biodegradable polymers	Chemical bond	Increased drug solubility, high drug-loading capability	Hydrolyzed easily

**Table 2:** The strategies of overcoming the transport barriers of NDDSs to codeliver two different drugs.

Delivery barriers	Physiological basis	Strategies	Feature	Nanocarrier type	Nanocarrier composition	Drug	Ref
(1) Blood circulation	(1) The mononuclear phagocyte system (MPS)	(1) Hydrophilic polymer nanoparticles	PEG-modified	Liposome	DSPE-PEG 2000	Adriamycin + quercetin	[42]
			PEG-modified	Micelles	mPEG-PCL	Doxorubicin + curcumin	[43]
	(2) Electrostatic interaction	(2) RBC cloak nanoparticles	PEG-modified	Nanoparticles	PEG-b-PLL	Doxorubicin + triptolide	[44]
			PEG-MSN	Mesoporous silica nanoparticle (MSN)	PEG-MSN	Paclitaxel + curcumin	[45]
			PEG-modified	Lipid-coated polymeric nanoparticle	PEG2k-DSPE/PLGA	Doxorubicin + curcumin	[46]
			PEG-MNPs	Magnetic nanocomposite	Fe3O4/HAPA/*β*-CD	Doxorubicin + curcumin	[47]

(2) Drug accumulation and penetrtion	(1) ECM	(1) EPR effect	RGD peptide-targeted	Liposome	DSPE-PEG 2000	Paclitaxel + curcumin	[48]
	(2) Vascular endothelial barrier	(2) Vascular targeting for accumulation	RGD peptide-targeted	Mesoporous silica nanoparticle	PAA-chitosan	Topotecan + quercetin	[49]
			RGD peptide-targeted	Lipid-coated nanoparticles	DSPE-PEG-NHS, PLGA	Sorafenib + quercetin	[50]
	(3) Thick stroma	(3) Antivascular targeting by inhibiting tumor angiogenesis	EPR	Liposome	SPC : Chol : DOPE/EPG	Doxorubicin + biochanin A	[51]
	(4) Proteolytic enzymes in the tumor		EPR	Lipid-polymer hybrid nanoparticles (Lpns)	DOPE/EPG	Cisplatin + curcumin	[33]
			Smaller and compacted scale nanoparticles	Self-assembled nanoparticle	PEG-VES	Sorafenib + curcumin	[52]

(3) Drug internalization into the targeted cells	(1) Electric interaction needed to stride over the cell membrane to enter inside the cells	(1) P-gp inhibitors combining nanoparticles	P-gp inhibitor (curcumin)	Amphiphilic polymeric micelle	PEG(2k)-PLA(5k)	Doxorubicin + curcumin	[53]
			P-gp inhibitor (tetrandrine)	Lipid polymer hybrid nanosystem	MAL-PEG-DSPE, PLGA	Paclitaxel + Tetrandrine	[54]
		(2) Receptor-targeted nanocarriers	Transferrin-targeted, pH-sensitive	Polymer-drug conjugate	Tf-PEG-CUR	Doxorubicin + curcumin	[55]
			FA-targeted	Planetary ball-milled (Pbm) nanoparticles	FA-PCL-PEG	Docetaxel + resveratrol	[56]
			FA-targeted	Lipid nanoparticles	GMS-TPGS-SA-FA	Paclitaxel + curcumin	[57]
			GLUT1-targeted	Polymeric micelles	PEG2000–DSPE	Doxorubicin + curcumin	[58]
			Transferrin-targeted	Polymeric micelles	PEG-PE	Paclitaxel + curcumin	[36]

(4) Drug release	(1) Acidic environment and specific enzymes present in CSCs in the tumor	(1) PH-sensitive nanoparticles	pH-sensitive	Micellar nanoparticles	DSPE-PEG-imine-MTX	Methotrexate + curcumin	[59]
			pH-sensitive	Nanoparticles	PEG-lipid/PAA/CaCO3	Doxorubicin + curcumin	[60]
		(2) Enzyme-sensitive nanparticles	pH-sensitive, CSCs-targeted	Micellar system	PPBV	Paclitaxel + curcumin	[61]
			pH-sensitive	Nanoparticle	TPGS-PAE	Doxorubicin + curcumin	[62]
		(3) Temperature-responsive	pH-sensitive	Copolymer	PCL-St-POX	Terminator + curcumin	[63]
			Pe-targeted, EPR	Peptosome	PePm/PS	Pe + curcumin	[64]
			Thermosensitive copolymer	Nanogels	Heparin-pluronic F127 (Hep-F127)	Cisplatin + curcumin	[65]
			Near-infrared (NIR)-responsive	Gold nanocages	Biotin-PEG-SH	Doxorubicin + quercetin	[66]

(5) Drug response	(1) Based on summarized pathophysiological basis	(1) Directly target and kill CSCs	pH-sensitive, CSCs-targeted	Polymeric nanoparticle	mPEG-PLGA-pglu	Doxorubicin + curcumin	[67]
			pH-sensitive, CSCs-targeted	Core-shell nanoparticle	VES-g-e-PLL/*γ*-PGA-Dopa	Doxorubicin + curcumin	[68]
		(2) Synergistic combination of two or more drugs	Combination of chemotherapeutic and plants extracts	Nanoliposomes	mPEG2000-DSPE, DOPA	Cisplatin + curcumin	[69]
				Phytosome	Quercetin and phospholipid (lecithin)	Doxorubicin + quercetin	[70]
				Lipid nanoparticles	Glyceryl distearate,triglycerides medium-chain, soybean lecithin/polyoxyl 40 hydrogenated castor oil, glycerin	Doxorubicin + curcumin	[71]
				Polymeric micelles	PCL-b-ABPA-b-POEGMEA	Platinum drugs + curcumin	[72]
				Nanoparticle	mPEG-PCL	Temozolomide + resveratrol	[73]
				Liposomal	Egg sphingomyelin/cholesterol/PEG2000 ceramide	Quercetin + vincristine	[74]
				Polymeric micelles	PGS2000/PEG2000-DSPE	Doxorubicin + curcumin	[75]
				Lipid nanoparticles	PEG-DSPE	Etoposide + curcumin	[76]
				Lipid-polymer hybrid nanoparticles (Lpns)	DSPE-mPEG_5000_/DSPE-PEG_5000_ FITC	Paclitaxel + triptolide	[77]
				Liposomes	Egg phosphatidylcholine/DSPE-PEG	Doxorubicin + resveratrol	[78]
				Nanoemulsion (NE)	PEG_400_-DOCA, HP-beta-CD	Pemetrexed + quercetin	[79]
				Bottlebrush copolymer-Based micelle	PEG-PNB-TC	Paclitaxel + curcumin	[35]
				Lipid-polymeric nanocarriers	PLGA, PEG_2000_-DSPE	Vincristine + quercetin	[80]
				Niosomes	Tween-60: cholesterol:DPPC : DOTAP : DSPE-PEG2000	Doxorubicin, quercetin + sirna	[81]
				Polymer-lipid nanoparticles	DSPE-PEG2000, POPC,DOPAC	Paclitaxel + curcumin	[82]
		(3) Multifunctional targeted delivery	pH-responsive, CRGDK-targeted, EPR	Nanoparticles	CRGDK-PEG-PCL	Doxorubicin + curcumin	[83]
			Magnetic-guided targeting, T7-mediated targeting	Nanoparticles	PLGA-PEG-T7	Paclitaxel + curcumin	[84]
			Lactoferrin- (Lf-) tethered magnetic-targeted	Magnetic micelle	PVA/PAA	Doxorubicin + curcumin	[37]
			pH-responsive, Folate receptor-targeted	Nanoplates	PEG methacrylate, PEG	Daunorubicin + curcumin	[85]
			EGFR peptide (GE11)-targeted, pH-sensitive, EPR	Prodrug nps	PLGA-PEG-Mal, PLGA-PEG-NH2, PEG-NH2	Docetaxel + curcumin	[86]
			Magnetic targeting, biotin receptors-targeted	Magnetic nanoparticles	Biotin-PEG-PCDA	Paclitaxel + curcumin	[87]

(6) Mutidrug resistance (MDR)	(1) Based on summarized pathophysiological basis	(1) Reverse transporter-mediated MDR (Inhibition of P-p, LRP, MRPs,BCRP)	PEGylation	Prodrug nanopaticle	PEG-curcumin	Docetaxel + curcumin	[88]
			PAMAM dendrimer	Copolymer nanopaticle	PEG-PAMAM	Paclitaxel + Borneol	[89]
			PEGylated	Liposome	DSPE-mPEG2000, PC	Paclitaxel + resveratrol	[90]
		2) Reverse apoptosis gene-mediated MDR	EPR	PLGA-lipid nanoparticles	DSPE-PEG2000, PLGA	Docetaxel + gambogic acid	[32]
			Anisamide- (AA-) targeted	Nanoparticles	PLGA, CHO-hyd-PEG-AA	Doxorubicin + resveratrol	[91]

(7) Immunoregulation	(1) Oxidative and enzymatic environment	(1) Anti-inflammatory effects	CD44-targeted	Nanohydrogel	FA-HA	Rapamycin + quercetin	[92]
			PEGylated	Long-circulating liposomes	DPPC, PEG-2000-DSPE	Docetaxel + curcumin	[93]

(8) Antagonize/reduce toxicity and side effects	(1) Reactive oxygen species (ROS) environment	1) ROS-sensitive nanoparticles	ROS-cavenger: curcumin	Polymeric micelles	mPEG-PCL	Docetaxel + curcumin	[94]

DSPE-PEG: 1,2-distearoyl-sn-glycero-3-phosphoethanolamine-*N*-[methoxy(polyethylene glycol); PLGA: poly(lactic-co-glycolic acid); mPEG-PCL: methoxy poly(ethylene glycol)-poly caprolactone; PEG-b-PLL: poly(ethylene glycol)-b-poly(L-lysine); HAPA: hydroxyapatite; *β*-CD: *β*-cyclodextrin; PEIIPDI-PEA: branched polyethylenimine-isophorone diisocyanate-poly(L-lactide)-PEI; PAA: polyacrylic acid; DSPE-PEG-NHS: distearoyl-L-a-phosphatidylethanolamine-polyethylene glycol-N-hydroxysuccinimide; SPC: soy phosphatidylcholine, Chol: cholesterol, EPG: egg phosphatidylglycerol, and DOPE: 1,2-dioleoyl-sn-glycero-3 phosphoethanolamine; PEG-VES: polyethylene glycol derivative of vitamin E succinate; MAL: maleimide; Tf: transferrin; FA: folate; GMS-TPGS-SA-FA: glyceryl monostearate-D-alpha tocopherol acid polyethylene glycol succinate-stearic acid and folate; PEG2000–PE: 1,2-distearoyl-sn-glycero-3-phosphoethanolamine-N-[methoxy(polyethylene glycol)-2000]; PPBV: poly(ethylene glycol)-benzoic imine-poly(gamma-benzyl-l-aspartate)-b-poly(1-vinylimidazole) block copolymer; TPGS-PAE: d-alpha-tocopheryl polyethylene glycol 1000-block-poly(beta-amino ester); PCL-St-POX: poly caprolactone-starch-poly(2-ethyl 2-oxazoline); PePm: TFIIATVEGVLLFLILVVVVGILIKRRGPLGVRGC, PS: peptosomes; mPEG-PLGA-PGlu: monomethoxy (polyethylene glycol)-b-P (D,L-lactic-coglycolic acid)-b-P (L-glutamic acid); VES-g-e-PLL: RRR-a-tocopheryl succinate-grafted-e-polylysine conjugate; *γ*-PGA-Dopa: poly-*γ*-glutamic acid-dopamine; DOPA: 1,2-dioleoyl-sn-glycerol-3-phosphate; PABPA: 3-((tert-Butoxycarbonyl)amino)propyl acrylate; POEGMEA: polymerisation of oligo(ethyleneglycol)methyl ether acrylate; DSPE-PEG5000-FITC: 1,2-distearoyl-sn-glycero-3-phosphoethanolamine-*N*-[(polyethylene glycol)-5000]-fluorescein isothiocyanate; DOCA: deoxycholic acid, HP-beta-CD: 2-hydroxypropyl-beta-cyclodextrin; PEG-PNB-TC: polyethylene glycol-polynorbornene-thiocresol; DPPC: 1, 2-dipalmitoyl-sn-glycero-3-phosphocholine phospholipid, DOTAP: 1, 2-dioleoyl-3-trimethylammonium-propane; POPC: 1-palmitoyl-2oleoyl-sn-glycero-3-phosphocholine; CRGDK: Cys-Arg-Gly-Asp-Lys; T7: sequence HAIYPRH.
